# Clinical characteristics, cause analysis and infectivity of COVID‐19 nucleic acid repositive patients: A literature review

**DOI:** 10.1002/jmv.26491

**Published:** 2020-11-01

**Authors:** Youjiang Li, Danping Ji, Wangyu Cai, Yingying Hu, Yongying Bai, Jianguo Wu, Jian Xu

**Affiliations:** ^1^ The Department of Clinical Laboratory, The Fourth Affiliated Hospital Zhejiang University School of Medicine Yiwu Zhejiang China; ^2^ The Department of Obstetrics and Gynecology Shangxi District Medical Community of Yiwu Central Hospital Yiwu Zhejiang China; ^3^ The Department of Obstetrics and Gynecology, The Fourth Affiliated Hospital Zhejiang University School of Medicine Yiwu Zhejiang China; ^4^ The Department of Clinical Laboratory, Women's Hospital Zhejiang University School of Medicine Hangzhou China

**Keywords:** COVID‐19, recovered patients, RT‐PCR test, SARS‐CoV‐2

## Abstract

Coronavirus disease 2019 (COVID‐19) poses a serious threat to human health and lives. The virus is still spreading throughout the world, and the cumulative number of confirmed cases is increasing. After patients with COVID‐19 are treated and discharged, some have repeated clinical symptoms and become positive for nucleic acid tests a second time. Through analysis and review of the existing literature, the proportion of repositive patients in the discharged patient population and their clinical characteristics were systematically described for the first time. Furthermore, an in‐depth analysis of the causes of repositive nucleic acid tests and the potential transmission of the disease provides the basis for the management and protection of discharged patients with COVID‐19.

## 1 INTRODUCTION

Coronavirus disease 2019 (COVID‐19) is caused by severe acute respiratory syndrome coronavirus 2 (SARS‐CoV‐2), which originated in Wuhan, Hubei Province, China, in December 2019, and is widespread globally.^1^, ^2^ According to information released by the World Health Organization (WHO), as of August 11, 2020, SARS‐CoV‐2 has spread to more than 200 countries worldwide, causing 19,718,030 infections and 728,013 deaths.^3^

Under the stringent scientific prevention and control measures employed by the Chinese government and people, the COVID‐19 epidemic has been effectively mitigated and controlled in China, and the number of recovered cases is increasing. Further monitoring of the disease prognoses and the use of effective control measures to prevent the recurrence of the epidemic have become the main focus of the country.

Currently, patients who have had COVID‐19, and were discharged from hospital after two consecutive negative nucleic acid tests for respiratory pathogens, are being readmitted as a result of being nucleic acid repositive on follow‐up visits. On February 27, 2020, the Journal of the American Medical Association, a top international journal, reported that four medical personnel who had been discharged from hospital after receiving treatment for COVID‐19 were found to have positive results on pharyngeal swab nucleic acid tests,^4^ which causes high levels of confusion and concern among members of the public and medical staff.

However, there is currently insufficient knowledge about the characteristics of repositive patients. In the manuscript, we reviewed the proportion, characteristics, potential reasons, and infectivity of repositive COVID‐19 patients to explain this phenomenon. In addition, suggestions for the prevention and control of viral reoccurrence in discharged patients are proposed.

### 1.1 The proportion and clinical characteristics of repositive patients

A large national study in South Korea identified that 292 (3.3%) out of 8922 recovered patients subsequently have at least one positive test postdischarge, however, does not describe if all recovered patients were tested or if only those cases who were symptomatic were tested postdischarge.^5^

In a follow‐up of 172 discharged patients, 11 patients were tested positive in nasal Swab and 15 in anal swab, with a positive rate of 14.5%. The average age of these 25 patients was 28 years old, and six of them were children under 12 years old, suggesting that nucleic acid repositive patients are generally younger than other patients. On first hospitalization, fever (68%) and cough (60%) were common symptoms, all of which were mild forms. At readmission, eight patients (32%) developed a mild cough.^6^ In a study by An et al.,^7^ 38 of the 226 discharged patients had nucleic acid reactivation with a positive rate of 16.8%. The repositve patients had significant characteristics of “double lightness.” When first admitted to the hospital, the patients' clinical symptoms were mild, and almost all of them were those who had had mild and common forms of the disease. Compared with the other discharged patients, their symptoms were less, and the RNA negative conversion time was earlier. Second, the repositive patients were young. Children younger than 14 years old accounted for 35% of the repositive patients, while there was only one repositive patient older than 60 years old. On the contrary, Xiao et al.^8^ reported that 15 repositive patients were older than the other 55 discharged patients and their nucleic acid conversion times may have been longer.

In the published literature, there are few of large‐sample statistics on the proportion of patients with viral redetectable, and the rate of viral redetectable has been reported to be as low as 3.3% and as high as 30.7% (see Table 1). However, the results of these small‐sample‐size studies suggest that a significant proportion of discharged patients are carriers of the virus. According to reports of the clinical characteristics of repositive patients, some patients have recurrent clinical symptoms, such as fever and cough.^6^, ^9^, ^10^, ^11^, ^12^ However, a large number of cases showed no clinical symptoms and no change in laboratory indicators or imaging findings.^3^, ^7^, ^8^, ^13^, ^14^, ^15^, ^16^, ^17^, ^18^ Furthermore, the repositive patients tended to be younger, and most of them had mild disease symptoms at the first time of admission.^6^, ^7^, ^13^, ^18^, ^19^

**Table 1:** Clinical characteristics of repositive COVID‐19 patients

	Yuan et al.^6^	An et al.^7^	Xiao et al.^8^	Deng et al.^10^	Wong et al.^11^	Zhu et al.^12^	Ye et al.^13^	Li et al.^14^	Cao et al.^15^	Yuan et al.^18^
Number of follow‐up cases	172	262	70	576	119	98	55	13	108	182
Number of repositives	25	38	15	61	21	17	5	4	8	20
Rate	14.5%	14.5%	21.4%	10.6%	19.8%	17.3%	9%	31%	7.4%	11%
Type of sample (type, no.)	Nasal swab, 11; anal swab, 15	Not mentioned	Throat swab or nasal swab	Nasal and throat swabs, 36; sputum, 8	Nasal swab	Sputum and nasal wab	Respiratory samples	Sputum	Nasal swab or throat swab	Throat swabs, 14; anal swabs, 6; both swabs, none
Severity of the disease (type, no.)	Moderate, 24; severe, 1	Mild, 11; general, 27	Moderate, 14; severe, 1	Mild, 38; general, 20; severe, 3	Asymptomatic, 3; mild, 13; moderate, 5	Not mentioned	Not mentioned	Moderate, 13	Moderate, 6; severe, 2	Mild and moderate, 20; severe, none
Average age, (range), (year)	Twelve cases were children under 14 years old (48%)	A significantly higher proportion of patients were children, and only one patient was over 60 years old	64 (51–73)	54.8	47	54 (44–63)	32.4（27–42）	52.8 (1–73)	54.37（26–72）	39.9 ± 20.1 (1‐72)
Sex	8 males and 17 females	Not mentioned	9 males and 6 females	25 males and 36 females	16 males and 9 females	5 males and 12 females	2 males and 2 females	6 males and 7 females	3 males and 5 females	7 males and 13 females
Date of admission	From Jan. 23, 2020 to Feb. 21, 2020	From Jan. 23, 2020 to Feb. 25, 2020	From Jan. 21, 2020 to Feb. 12, 2020	Not mentioned	Not mentioned	Not mentioned	From Jan. 8, 2020 toFeb. 10, 2020.	From Jan. 26, 2020 to Feb. 6, 2020	From Feb. 10, 2020 to Apr. 13, 2020	Not mentioned
Time from discharge to repositive (day)	7.32 ± 3.86	Not mentioned	Not mentioned	10 (3–35)	11–18	4（3–8.5）	10.7 (4–17)	5–14	Not mentioned	7–14
Complication	No	Not mentioned	Not mentioned	Eight cases of chronic obstructive pulmonary disease, five cases of hypertension and 5 case of diabetes	Not mentioned	Two cases of hypertension and one case of diabetes	No	Four case of hypertension and one case of chronic lymphocytic leukemia	One case of hypothyroidism and one case of preexisting pulmonary tuberculosis	Not mentioned
Clinical characteristics on first admission	Fever (68%) and cough (60%) were predominant; mild and common forms were predominant, and only one case was severe	Clinical symptoms were mild and normal. Fewer symptoms than other discharged patients. CT image relief was more persistent and RNA negative conversion time was earlier	Not mentioned	Not mentioned	Not mentioned	There was no significant difference of initial clinical symptoms between repositive group and nonrepositive group.	One patient had no symptoms and four had mild symptoms (mainly fever and cough)	All patients presented with the common symptoms of fever, cough, fatigue, muscle soreness, and sore throat.	The first symptoms were fever, cough, and sore throat. The clinical features were not significantly different from those of other patients	Tere were no signifcant diferences between re‐positives and non‐re‐positives in terms of age median, sex, and comorbidities
Clinical characteristics on second admission	At readmission, eight patients (32%) developed a mild cough	No clinical symptoms; laboratory and CT findings were normal	No clinical symptoms; laboratory and CT findings were normal	38 (62.3%) patients were asymptomatic. The most common symptoms were fever (24.6%), cough (18%), sputum production (14.8%), and sore throat (13.1%).	20 (95%) of the re‐positive patients were asymptomatic. Only one reported symptoms postdischarge, that is a mild cough.	One case showed exudative lesion recurrence in pulmonary computed tomography (CT) with recurred symptoms	No clinical symptoms; laboratory and CT findings were normal	No clinical symptoms; laboratory and CT findings were normal.	No clinical symptoms; laboratory and CT findings were normal	No clinical symptoms; laboratory and CT findings were normal

Abbreviations: COVID‐19, coronavirus disease 2019; CT, computerized tomography.

Compared with other discharged patients, no clinical characteristics or indicators were found to reliably predict the risk of a patient being repositive for SARS‐CoV‐2, nor were any specific drugs or treatments associated with SARS‐CoV‐2 reactivation. However, whether there are clinical symptoms or not, such patients are admitted to hospital, provided medicine treatment, monitored for physical changes, and regularly tested for nucleic acid conversion (see Table 1).

### 1.2 Cause analysis for recovered COVID‐19 patients with repositive nucleic acid results

In the existing literature, strict isolation measures continue to be taken for discharged patients, and the chance of reexposure to the source of infection is very low,^7^, ^8^, ^11^, ^17^ which suggested that nucleic acid repositive patients are not reinfected with SARS‐CoV‐2, rather, it is likely that the virus was not completely cleared before the patients were discharged from hospital, and their previous nucleic acid test results were false negative. To ascertain why the nucleic acid results were false negative and SARS‐CoV‐2 is not easily cleared, the etiological and patient characteristics and laboratory tests should be analyzed in combination with the relevant literature.

### 1.3 Biological characteristics of SARS‐CoV‐2

Since the outbreak of SARS in 2003 and MERS in 2012, the possibility of coronaviruses spreading from animals to humans has been confirmed.^20^, ^21^ SARS‐CoV‐2 is similar to some of the coronaviruses detected in bats but distinct from SARS‐CoV and MERs‐CoV. Its conserved replicase domain (ORF1a/b) has less than 90% nucleotide sequence similarity with other β‐coronaviruses, thus, it is a new type of coronavirus.^22^, ^23^, ^24^, ^25^

Shen et al.^26^ recently found a significant level of viral diversity in some infected patients, suggesting the rapid evolution of SARS‐COV‐2. During the rapid development of the epidemic, the virus was prone to point mutations during human‐to‐human transmission. In a latest study, the researchers collected 48,635 SARS‐CoV‐2 complete genomes from the GISAID consortium and thousands of contributing laboratories. All SARS‐CoV‐2 mutations were analyzed and annotated compared with the reference Wuhan genome NC_045512.2, observing an average of 7.23 mutations per sample, and the prevalence of single nucleotide transitions as the major mutational type across the world.^27^ The SARS‐CoV‐2 genome contains 5′‐end replicase encoding genes (open reading frame 1AB, ORF1ab) and structural protein‐encoding genes (spike protein, S; envelope protein, E; membrane protein, M; and nucleocapsid protein, N).^28^ If a mutation site is in the primer binding area for nucleic acid amplification, false‐negative test results may occur. During the development of the kit, China CDC recommended that ORFlab and N genes be tested (most of the previous companies included E genes). The detection region of WHO was RdPP and E genes, and the detection region of the American CDC was N genes. Amplification of multiple gene regions and selection of the optimal probe combination by sequence alignment can effectively avoid the influence of nucleic acid variation on the detection results.

There is no definite literature to confirm the influence of variation on the test results, and the influence of virus variation on the test results belongs to theoretical reasoning. Due to the lack of a comprehensive, in‐depth understanding of SARS‐COV‐2, there is no specific drug for SARS‐COV‐2. The patient's immunity to the virus is mainly dependent on the patient's immune system, which results in the virus persisting in the patient's body. Therefore, relapse and migration may be characteristics of the new virus.

### 1.4 The duration of infection and condition of the patient

Most patients infected with SARS‐COV‐2 experience an initial asymptomatic incubation period, followed by mild, severe, and symptomatic remission.^29^ The viral load may differ between patients with different disease durations. The outcome of a patient's illness is often affected by many factors. Zheng et al.^30^ studied the results of continuously monitoring the nucleic‐acid load of 96 patients with COVID‐19 and found that the rate of positive respiratory nucleic acids gradually decreased from 94% in the first week of symptoms to 56% after 4 weeks; while the rate of positive fecal and serum samples gradually increased from the first week to the third week. The nucleic acid load in severely ill patients was significantly higher than that in mild patients. According to the current situation, pulmonary inflammation in some patients does not achieve full clinical recovery during the absorption process, and there may be intermittent detoxification phenomena, leading to positive nucleic acid tests after discharge.

### 1.5 Patient's immunity

The reoccurrence of positive COVID‐19 results may be related to a patient's immunity, as a decline in immunity easily leads to a resurgence in the body's viral load and disease relapse. In an early COVID‐19 study, the blood antibody tests of 16 patients with COVID‐19 showed positive immunoglobulin M (IgM) and immunoglobulin G (IgG) rates of 50% (8/16) and 81% (13/16) on Day 10 of infection and rates of 81% (13/16) and 100% (8/16) on Day 15, respectively.^31^ Another study on SARS showed that anti‐SARS‐CoV IgG can last up to 12 years in the human body.^32^ These studies suggest that after recovery from SARS‐CoV‐2, patients may carry protective antibodies and maintain their immunity for a long time, but the production of antibodies does not necessarily mean that the patient will not be reinfected. Bentivegna et al.^33^ describe a case of a patient recovered from COVID‐19 pneumonia with positive serology, followed up by six negative nasopharyngeal swab RT‐PCR tests performed along 1 month who later on, after exposure to the virus, presented another positive reverse transcriptase RT‐PCR test and a second IgM seroconversion. Which suggested that the patient was reinfected.

Guo et al.^34^ found that absolute counts of CD3+ T cells, CD3+CD4+ T cells, and CD3+CD8+ T cells were significantly reduced in patients affected by viral pneumonia death. Therefore, the decreased cellular immune function may affect the development of the disease, which may be related to nucleic acid reactivation in discharged patients. Liu et al.^35^ found that albumin is an independent risk factor for the progression of COVID‐19. The immune system defends against external infections by providing tools, such as white blood cells, lymphocytes, interleukins, and so forth, which help to remove foreign microorganisms and fight viruses. In the existing reports of nucleic acid repositive patients, children and older patients with low immunity are more likely to have difficulties clearing the virus and test positive after discharge than middle‐aged patients.^6^, ^13^, ^19^

### 1.6 The nucleic acid test was false negative before discharge

#### 1.6.1 The sampling site

Whether the test results accurately reflect the true viral load of patients is dependent on the collection of suitable samples. The respiratory secretions sampled mainly consist of nasopharyngeal swabs, oropharyngeal swabs, sputum of the lower respiratory tract, and alveolar lavage fluid. The rate of positive nucleic acid tests varies because of the different viral contents in the different samples. Yang et al.^36^ analyzed 866 respiratory tract samples from 213 patients with COVID‐19, including 205 oropharyngeal swabs, 490 nasopharyngeal swabs, 142 sputum samples, and 29 alveolar lavage samples. For patients with severe and mild symptoms, the rate of positive results was significantly higher for sputum (88.9% and 82.2%, respectively) than for nasopharyngeal swabs (73.3% and 72.1%, respectively). The rate of positive results from oropharyngeal swabs was the lowest (60.0% and 61.3%, respectively), while the positive result rate for alveolar lavage fluid collected from severe patients was as high as 100%. Zhou et al.^37^ collected oropharyngeal and nasopharyngeal swabs from 17 patients with obvious symptoms and found that the nucleic acid load for novel coronavirus was significantly higher in the nasopharyngeal swab than the oropharyngeal swab. The main area of SARS‐COV‐2 infection is the lower respiratory tract, and alveolar lavage fluid or deep sputum are the most suitable samples.^38^ However, most COVID‐19 patients have no sputum, and bronchoscope lavage is invasive; therefore, lower respiratory tract specimens are difficult to obtain. Currently, nasopharyngeal and oropharyngeal swabs are mainly collected clinically. False‐negative results may occur when upper respiratory tract samples are taken when the viral load is reduced after the patient enters the convalescence period.

#### 1.6.2 Sampling and testing methods

How closely the collection, preservation, and inspection methods follow the strict recommended standards will affect the quality of the specimens and, ultimately, the accuracy of the nucleic acid test results. In a comparative study of confirmed patients, the nucleic acid detection rate for common swabs was significantly lower than that of flocking swabs using virus preservation solution.^39^ Sampling materials, volume, timing, and operation will all affect the test results, for example, throat and nasal swabs are sampled by hand and difficult to standardize. When medical workers who are unskilled or have insufficient training collect samples, patients may react negatively, leading to an incorrect sampling position, inadequate sampling depth and time, and insufficient virus collection and resulting in false‐negative results. Additionally, if the samples are not sent for testing quickly or the storage solution is not used properly, the nucleic acids will rapidly degrade, which is also an important factor in false‐negative nucleic acid tests.

#### 1.6.3 Reagent performance

Because of the sudden outbreak of COVID‐19, there are a large number of urgent clinical needs. After the SARS‐COV‐2 genome was sequenced, researchers around the world established nucleic acid detection systems based on the sequence. To respond quickly and effectively to the epidemic, countries have accelerated the development and approval of SARS‐COV‐2 nucleic acid testing products. The quality of the reagent kits from busy reagent manufacturers may vary, there is no time for comprehensive performance validations with numerous clinical samples, and their stability and reliability need to be further confirmed. Different reagent brands have different detection limits. Most of the patients in the rehabilitation period are in the recovery stage of self‐limited disease, and the virus in the body may not be completely cleared. The amount of virus collected in the relevant parts is too small to detect the virus, which is lower than the detection limit of the kit. A laboratory^40^ have evaluated the consistency of the test results and detection ability of five nucleic acid testing kits approved by the National Medical Products Administration and two unlisted testing kits. Positive samples from 10 confirmed patients were tested, and the positive detection rate of six of the reagents was 100%, while that of the seventh reagent was only 80%. After the continuous dilution of samples, the seven reagents showed significantly different abilities to detect weakly positive samples (4 times and 16 times diluted samples). An et al.^7^ retested 24 samples that tested negative with commercial kits with a highly sensitive reagent, and 18 of the samples tested positive. These findings suggest that the sensitivities of the existing commercial reagents are still inadequate for clinical needs. For weakly positive samples, the possibility of false‐negative results is high. Therefore, some highly suspected patients are still negative for SARS‐COV‐2 in multiple clinical tests. In these cases, the use of more than two reagents is recommended for testing and verification.

#### 1.6.4 The virus is in other tissues

In early reports, 2–10% of patients with COVID‐19 had gastrointestinal symptoms such as diarrhea, abdominal pain, and vomiting.^41^, ^42^ Recent evidence has revealed that nucleic acid was redetected in stool swabs and feces of patients with COVID‐19.^43^, ^44^ Wang et al.^45^ reported three discharged patients who were readmitted due to obvious gastrointestinal symptoms and continued positive fecal nucleic acid. This demonstrated that the digestive system may have been the main target organ of SARS‐CoV‐2 in the three Patients. Based on the above information, the virus may remain in the small intestine, and from there reinfects the host. So the management and detection of feces of discharged patients should be strengthened.

### 1.7 The infectivity of nucleic acid repositive patients after hospital discharge

The infectivity of nucleic acid repositive patients is rarely reported. In the existing literature, there have been no reports of recovered COVID‐19 patients infecting other people, and some patients are nucleic acid negative again within a short time. PCR, which has been used in the clinical laboratory for over 30 years, has become the preferred method for nucleic acid detection in vitro. However, an important limitation of PCR detection is that it does not distinguish between viral replication and noninfectious nucleic acid residues. When studying Ebola, researchers found viral RNA in stool samples after the virus had cleared from the blood of recovered patients, but attempts to maintain the virus in cell culture were unsuccessful.^46^, ^47^ A research team in Shenzhen, China, found no positive nucleic acid results or clinical symptoms related to pneumonia during a 14‐day follow‐up of 21 close contacts of 38 patients who were nucleic acid repositive after discharge.^7^ In another report, out of 111 close contacts tested, none were found to be positive as a result of exposure to a repositive patient.^11^ However, the above reports were limited to a small group of people, the observation and follow‐up times were not long enough, and there was no clear evidence confirming whether the nucleic acid repositive patients were infectious. Because the detection limit of the reagent, sampling site, and sampling method has a substantial impact on nucleic acid test results, many of the nucleic acid repositive results may have been false negative.^36^, ^37^, ^39^, ^40^ Some patients still have a fever, coughing, and other symptoms after discharge, and even computerized tomography (CT) results are repeated, indicating that the virus still replicates in some patients, and they are more likely to be infectious. Therefore, considering that there is no clear evidence to disprove that repositive patients can not transmit the disease, they should receive scientific follow‐up and conform to strict isolation after discharge to avoid the risk of disease transmission.

### 1.8 Prevention and control suggestions for discharged patients with COVID‐19

According to published studies, the rate of nucleic acid reactivation in discharged patients is not low. In view of this, medical institutions should strictly follow up the discharged patients to monitor for disease recurrence. We make two Suggestions. First, all discharged patients should be isolated for at least 2 weeks. To prevent cross‐infection caused by home isolation, it is better to have centralized isolation, such as hotel or hospital isolation, when conditions permit. Second, there should be regular follow‐up visits for the first, second, and fourth weeks after discharge. Respiratory nucleic acid tests, and chest CT examination should mainly be reviewed during the reexamination. Patients with incomplete chest CT absorption should be followed up until the CT results are completely normal. If nucleic‐acid positive or CT‐progressive patients are found, prevention and control management should be carried out according to the source of infection. Such patients should be admitted to hospital, quarantined, and centrally treated.

### 1.9 Conclusion and prospects

A large proportion of patients who have recovered from COVID‐19 are nucleic acid repositive after discharge from hospital. Most of the cases reported in the literature did not present clinical symptoms or CT findings. However, some patients still had symptoms, such as fever, cough, and fatigue, and there were no reliable clinical features or indicators to predict the risk of being re‐positive for SARS‐CoV‐2. Although there have been no reports of discharged patients infecting others, there is no conclusive evidence that they are not contagious. The occurrence of false‐negative nucleic acid tests should be minimized by careful optimization of specimen collection and test reagent performance. At the same time, the follow‐up management of discharged patients should be improved, the virus status of patients should be monitored regularly, and, for patients testing nucleic acid positive a second time, centralized and isolated care and treatment should be provided. The causes and mechanisms of the phenomenon of repositive results, as well as the infectiousness of these patients, still needs further research and exploration by scientists around the world.

## CONFLICT OF INTERESTS

The authors declare that there are no conflict of interests.

## AUTHOR CONTRIBUTIONS

All authors contributed to the conception of the Review. Youjiang Li, Danpi Ji, Yingying Hu and Yongying Bai contributed the literature search, data traction and data synthesis, created the tables, and wrote the manuscript. Jian Xu and Jianguo Wu critically reviewed and edited it with their comments.

## ETHICS STATEMENT

This study was reviewed and approved by the Medical Ethical Committee of The Fourth Affiliated Hospital, Zhejiang University School of Medicine.
